# Comparison of GO/g-C_3_N_4_ nanocomposite with g-C_3_N_4_ light-activated humidity sensors at room temperature

**DOI:** 10.1038/s41598-026-52607-3

**Published:** 2026-05-09

**Authors:** Nafiseh Tobeiha, Nafiseh Memarian, Fatemeh Ostovari

**Affiliations:** 1 Faculty of Physics, Semnan University, Semnan, Iran; 2 Department of Physics, Faculty of Science, Yazd University, Yazd, Iran

**Keywords:** g-C_3_N_4_, GO/g-C_3_N_4_ nanocomposite, Humidity sensor, light-activated sensor, Chemistry, Materials science, Nanoscience and technology, Optics and photonics

## Abstract

Numerous efforts, have been made to achieve higher efficiency in humidity sensors through the use of nanomaterials. Among these, two-dimensional nanomaterials have played a prominent role in enhancing the performance of such sensors. The utilization of various lasers operating at different wavelengths plays a pivotal role in activating humidity-sensing mechanisms, and the synergy between relative humidity (RH) and light irradiation leads to amplified sensing responses in nanomaterials. In the present research, g-C_3_N_4_ nanosheets and GO/g-C_3_N_4_ nanocomposite were prepared by sonochemical exfoliation. The physical properties of these 2D nanomaterials were investigated using different characterization techniques. Subsequently, humidity sensors based on these materials were fabricated on Si/SiO_2_ substrates. The study compared the humidity sensing capabilities of g-C_3_N_4_ nanosheets and GO/g-C_3_N_4_ nanocomposite at dark conditions, as well as when exposed to laser light at different wavelengths. The results indicate that the light-activated humidity sensor made from GO/g-C_3_N_4_ nanocomposite performs better under 660 nm laser irradiation across different RHs, compared to other wavelengths. Furthermore, under light irradiation, the response and recovery times of the nanocomposite were obtained as 0.5 s and 1 s, respectively, indicating superiority irradiation of nanocomposite under 660 nm wavelength in different RHs of 40% and 80% compared to the dark condition. This pioneering study marks the first empirical validation that the GO/g-C_3_N_4_ nanocomposite sensor surpasses its pristine g-C_3_N_4_ counterpart for light-activated humidity-sensing applications.

**Supplementary Information:**

The online version contains supplementary material available at 10.1038/s41598-026-52607-3.

## Introduction

Humidity sensors have applications beyond environmental monitoring; they are essential for numerous applications in meteorology, agriculture, and industrial uses, particularly in pharmaceuticals and food processing, where even a minor discrepancy can lead to degradation of the products due to moisture absorption^1,2^. Additionally, these sensors facilitate the optimal enhancement of crop-growing conditions by measuring soil moisture content and greenhouse climate, thereby improving both production quality and sustainability^3^. This remarkable transformation not only underscores the necessity of humidity sensors across various fields but also highlights their significance in maintaining product integrity and operational efficiency^4^.

Researchers are continuously investigating new materials, such as metal-halide perovskites^5^ and combinations of metal oxides^6^ with hybrid materials like graphene^7^, polymers^8^, and carbon materials^9^, which are predicted to offer better sensitivity and response time, potentially revolutionizing the way humidity is managed in industries^10^. Recently, two-dimensional (2D) materials, such as graphene, MXenes, transition metal chalcogenides, and g-C_3_N_4,_ have been widely endorsed by scientists as sensor materials for a wide range of sensors, such as pressure, humidity, sound, and gas sensors^11^.

Carbon nitrides (C_3_N_4_) have attracted considerable scientific attention over the past few decades. In 2009, Wang et al. discovered a metal-free compound called graphitic carbon nitride (g-C_3_N_4_), which has shown promising photocatalytic properties^12^. As a polymer-based material, it mainly comprises C and N atoms^13,14^. A few reports showed that it is possible to replace N atoms with C atoms, leading to non-local π bonds. Therefore, the electrical conductivity of g-C_3_N_4_ can be enhanced by delocalized π bonds that facilitate electron transfer^15,16^.

Carbon is a material that is found everywhere, while graphene (G), the epoch-making material of the modern age, is also an allotrope of carbon. Graphene is a 2D, single-layer sheet of sp^2^ hybridized carbon atoms and has attracted much attention and research motivation due to its versatile properties^17,18^. Graphene oxide (GO) has the same hexagonal carbon structure as graphene but also contains hydroxyl (–OH), alkoxy (C–O–C), carbonyl (C–O), carboxylic acid (–COOH), and other oxygen-based functional groups. Apart from the ease of synthesis, these oxygenated groups have many advantages over graphene, including higher solubility and the possibility of surface functionalization, which have offered many opportunities for use in nanocomposite materials^19^.

Some researches have been studied the humidity sensors based on carbon materials. For instance, J. Kang et al. investigated flexible, multilayer humidity sensors based on lysine-modified GO/ g-C_3_N_4_ composites (GO-Lys). The GO/g-C_3_N_4_ sensor exhibited a sensitivity of about 2.41 log(RH%)/%RH with a response/recovery time of 12.8 s. The ion enhancement, effected by the addition of lysine (GO-Lys), and under visible light irradiation. This study represents an advance in multilayer humidity electronics, and light-sensitive sensing for human-oriented applications such as breath analysis and wearable trackers^20^. Another research investigated the sensing capabilities of a g-C_3_N_4_/GQDs humidity sensor in a wide range of humidity levels, from 7% to 97% RH. The results demonstrated excellent reversibility and fast response and recovery times^21^. In another study, Debasree Burman et al. investigated a humidity sensor based on MoS_2_/GO nanocomposite. They believed that the high response was due to proton conductivity in the water layer for MoS_2_ and GO. The sensor’s performance remained consistent even after three months, demonstrating its excellent repeatability. Additionally, it exhibited strong sensitivity, as evidenced by its quick response time and efficient recovery^22^. Recently, Wang et al. investigated a nanohybrid humidity sensor of tin oxide (SnO_2_)/graphitic carbon nitride (g-C_3_N_4_) enriched with oxygen vacancy (O_v_) and –NH_x_ functional groups, This hybrid overcomes the degradation of g-C_3_N_4_ at high humidity by preventing the separation of the hydrophilic layer, while the O_v_ sites enhance water absorption and proton conduction. Long-term tests demonstrate negligible performance decay, highlighting the organic-inorganic synergy for durable operation^23^.

The present research investigated the impact of laser light radiation with different wavelengths on humidity sensors made from g-C_3_N_4_ and GO/ g-C_3_N_4_ nanocomposite. Our initial approach involved preparation and characterization of the g-C_3_N_4_ and GO/g-C_3_N_4_ nanocomposite materials. Next, for the first time, we compared the performance of the g-C_3_N_4_ with nanocomposite humidity sensors in both dark condition and under laser light radiation with wavelengths of 450 and 660 nm.

##  Experimental

### Synthesis and materials characterization

g-C_3_N_4_ was prepared using the thermal decomposition method^24^. Typically, 3 g of urea was placed into an alumina crucible and then heated in a muffle furnace at a temperature of 600 $\:\mathrm{℃}$ for 2 h at a heating rate of 3̊ C/min. Finally, the material was cooled to room temperature at 5 °C/min.

Graphene oxide (GO) was synthesized by exfoliation from the liquid phase of graphite oxide. Graphite oxide was also prepared by Hamer’s method^25^. 5 mg of graphite oxide powder was placed in 100 mL of deionized water for 3 h in an ultrasonic bath. Finally, it was washed with deionized water and ethanol in a centrifuge at 12,000 rpm and dried in an oven at 60 °C for 24 h to obtain GO.

For the synthesis of GO/g-C_3_N_4_ nanocomposite, First, 50 mg of GO powder was placed in 75 mL of a 55% ethanol to 45% deionized water solution on a magnetic stirrer and then subjected to an ultrasonic bath for 3 h. After that, we combined 50 mg of g-C_3_N_4_ with 75 mL of deionized water and ethanol, and followed the same procedure as above. Then, after 24 h of rest, both solutions were combined and placed in an ultrasonic bath for 3 h. Finally, the obtained solution was placed in an oven at 60 °C for 24 h to obtain the resulting powder.

The morphological features of the samples were carried out using field emission scanning electron microscopy (FESEM, MIRA3 TESCAN). Also, The elemental composition of samples was found out by energy-dispersive X-ray spectroscopy (EDX) integrated with the FESEM facility. The crystalline structure of the samples was determined by X-ray diffraction (XRD, Bruker Advance D-8) employing CuKα radiation (λ = 1.54 Å). Fourier-transform infrared (FTIR) spectra were recorded using a Shimadzu 8400 S spectrometer, with the samples prepared in KBr pellets. In addition, the optical properties were studied using Photoluminescence spectroscopy (PL, Shimadzu RF-6000 ), and diffuse reflectance spectroscopy (DRS) was employed using an Avantes AvaSpec 3648 spectrophotometer.

### Sensor analysis setup

Light-activated humidity sensors of g-C_3_N_4_ nanosheets and GO/g-C_3_N_4_ nanocomposites were fabricated as follow: at first the active material was deposited on a silicon substrate by dip coating. Then, by appling the silver paste ohmic contact were placed at the corners of the silicon piece to have a sensor with dimension of 0.5 cm^2^.

Humidity sensing experiments were carried out in a humidity chamber and cloud simulator at 23 °C (room temperature). To study the light-activated sensing behaver of samples, laser source have been applied. The laser source fabricated by Iran Biophotonic Technology Group, including low-power diode lasers for the medical applications, have wavelength capabilities of 400 to 1000 nm.

## Result and discussion

**Fig. 1:**
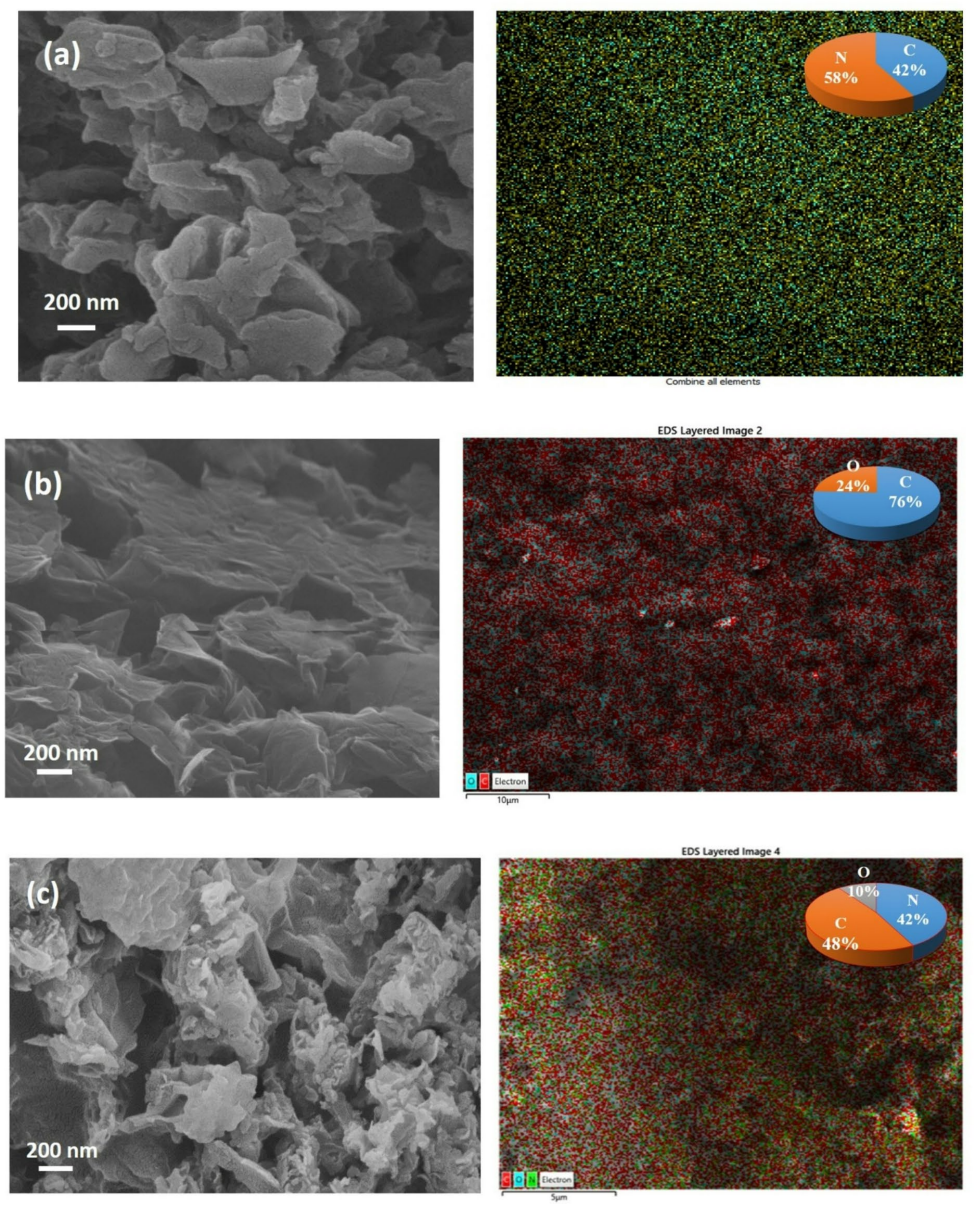
FESEM and EDS spectra of (**a**) g-C_3_N_4_, (**b**) GO, and (**c**) GO/g-C_3_N_4_ nanocomposite samples.

Figure 1 shows the FESEM images and EDS map spectra of the g-C_3_N_4_ (Fig. 1a), GO (Fig. 1b), and GO/g-C_3_N_4_ nanocomposite (Fig. 1c) samples. The nano-sheet like structure can be observed for all the samples. Also, Table 1 indicates EDX analysis of three samples. In the EDX analysis, the atomic ratio C/*N* = 0.62 for g-C_3_N_4_, the ratio C/O = 2.34 for GO. The EDX analysis, GO/g-C_3_N_4_ nanocomposite, shows the atomic ratio C/*N* = 0.88 and C/O = 4.25.

**Table 1:** EDX data of (a) g-C_3_N_4_, (b) GO, and (c), GO/g-C_3_N_4_ nanocomposite samples.

Atomic (%)	g-C_3_*N*_4_	GO	GO/g-C_3_*N*_4_
C	42.00%	75.73%	42.28%
N	58.00%	–	47.77%
O	–	24.27%	9.94%
Total	100%	100%	100%

**Fig. 2:**
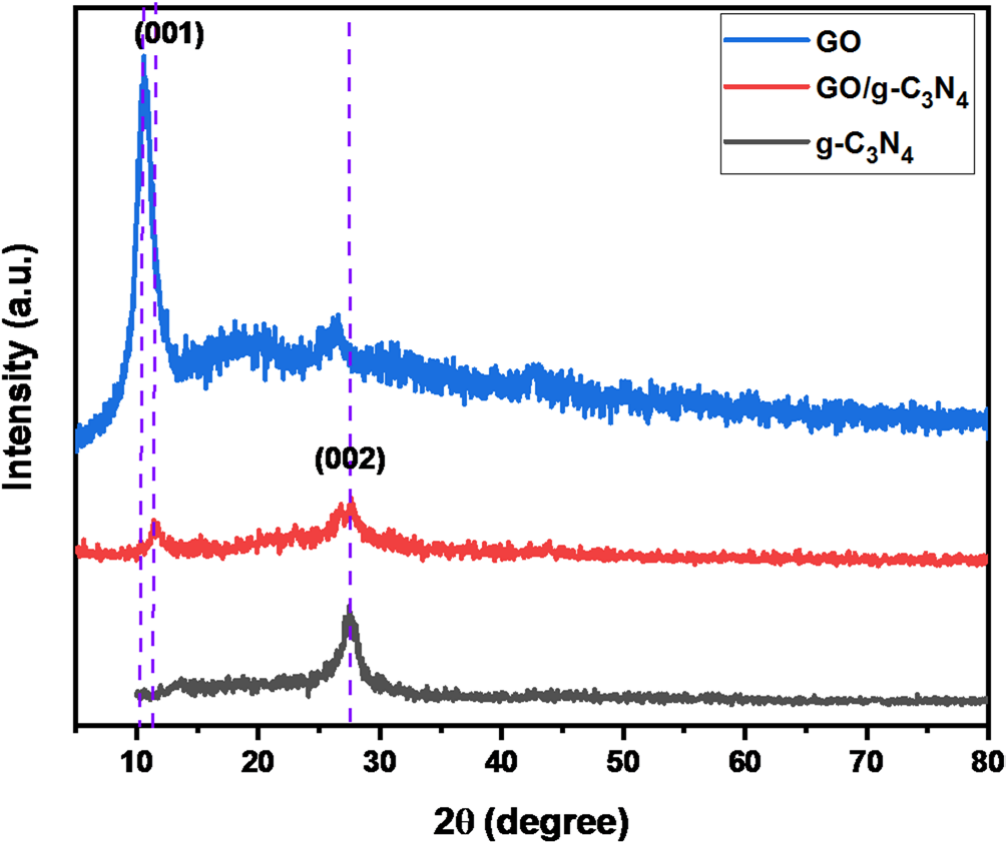
XRD patterns of GO, g-C_3_N_4_, and the GO/g-C_3_N_4_ nanocomposite.

The XRD patterns of the samples are presented in Fig. 2. The XRD patterns of the synthesized nanomaterials were authenticated by referencing JCPDS cards as #00-041-1487 for GO, #96-047-1526 for g-C_3_N_4_, and #01-87-1526 for the GO/g-C_3_N_4_ nanocomposite. The peak at 2θ = 10.6° is attributed to (001) plane of GO structure^22^. For g-C_3_N_4_ structure, a peak at 2θ = 27.2° is observed, which is corresponding to the (002) plane of g-C_3_N_4_, signifying the aggregated structure of the triazine rings. In the GO/g-C_3_N_4_ nanocomposite structure, peaks appear at 2θ = 11.4° attributed to GO and 2θ = 27.6° associated with the g-C_3_N_4_ componentwhich shows shifting to higher reflection angles due to the changes in the interlayer spacing in the nanocomposite structure^26^. The interlayer spacing was obtained using Bragg’s law:

1 $$\:\mathrm{n}{\uplambda\:}=2\mathrm{d}\mathrm{sin}{\uptheta\:}$$

where n is the order of reflection, λ is the wavelength of the X-ray, d is the interlayer spacing, and θ is the reflection angle. For GO d_001_ = 0.57 nm, and for g-C_3_N_4_ interlayer distance was calculated as d_002_ = 0.327 nm. However, in the GO/g-C_3_N_4_ nanocomposites the interlayer distances were obtained as d_001_ = 0.77 nm which shows expansion of interlayer distance for GO structure, and d_002_ = 0.322 nm which shows a very slight compertion for g-C_3_N_4_ in the nanocomposite structure, which affects the material’s crystalline quality and particle size^26,27^.

**Fig. 3:**
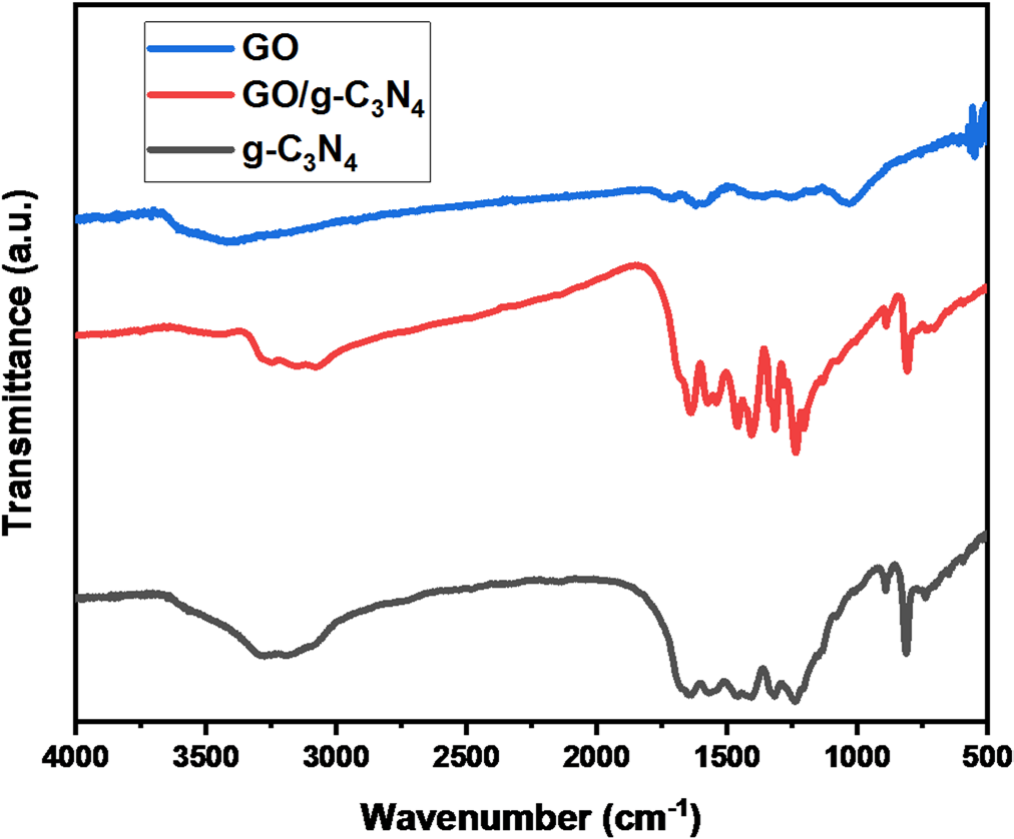
FTIR spectra of g-C_3_N_4_, GO, and GO/g-C_3_N_4_ nanocomposite.

The FTIR spectra of the samples are shown in Fig. 3. Furthermore, Table 2 shows the FTIR results interpretation of all three samples. Peaks @ 1639 cm^− 1^ and 1575 cm^− 1^ are attributed to stretching vibrations of C =C bonds, which are observed for both GO and nanocomposite samples^21^. The peaks around 1320 cm^− 1^ are related to out-of-plane bending vibrations of heptazine rings in g-C_3_N_4_ structure^12^. Peaks between 3000 and 3263 cm^− 1^ indicate the stretching mode of N–H bonds and absorption of water molecules^28^. As it is clear, the FTIR results support the synthesis of the GO/g-C_3_N_4_ nanocomposite.

**Table 2:** FTIR results of the GO, g-C_3_N_4_, and GO/g-C_3_N_4_ samples.

Wave number (cm^− 1^)										References
Sample	3000–3600	1782 & 1714 & 1382	1645 & 1636 & 1575	1650	1598	1319 & 1236 & 1354	1050	1037	891–808	
GO	–OH	–C–OH	C=C		–C–OH	–	–	C–O	–	^29–31^
g-C_3_N_4_	–NH	–	–	–C–N	–	out-of-plane bending vibrations of heptazine rings	–C–N	–	Tri-triazine ring	^24^
GO/g-C_3_N_4_	–NH & –OH	–	C=C	–C–N	–	out-of-plane bending vibrations of heptazine rings-C–N	–C–N	–	Tri-triazine ring	^21,24,28,32^

**Fig. 4:**
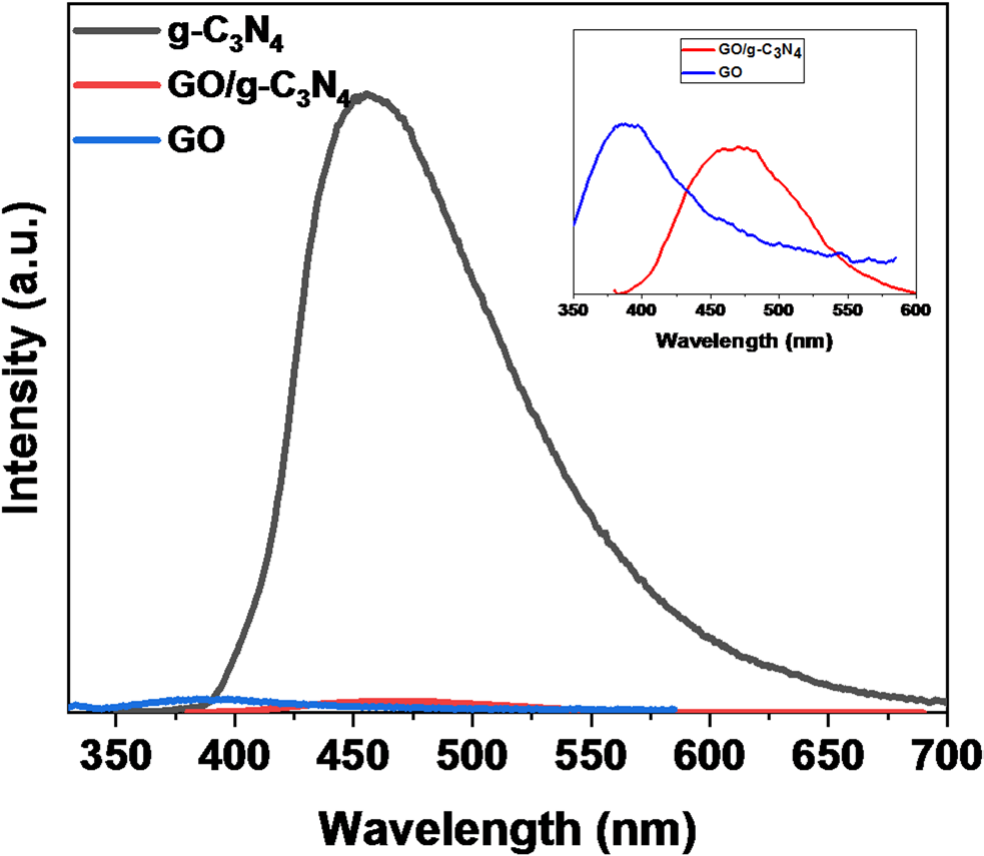
PL of g-C_3_N_4_,GO, and GO/g-C_3_N_4_ samples, inset shows the magnification of GO and GO/g-C_3_N_4_ samples.

The PL spectra of the samples are presented in Fig. 4. As can be seen, the intensity of the emission peak for the g-C_3_N_4_ sample is much more than for the GO and nanocomposite samples. The inset shows a magnified view of those two samples. The excitation wavelength for the GO sample was 300 nm, and a strong emission peak located at 386 nm can be observed, which is caused by electron transfer from carbon to oxygen due to the bonding between oxygen atoms and π states near the Fermi surface of graphene oxide. Furthermore, this peak is caused by the radiative recombination of electron-hole pairs located in sp^2^ groups^32–34^. The excitation wavelength for the g-C_3_N_4_ sample was 320 nm, indicating a peak at 456 nm, which is associated with the π*-π transition of the C-N sp^2^ bond^24^. The PL excitation wavelength for the GO/g-C_3_N_4_ nanocomposite was 360 nm. The results revealed a peak at 469 nm in the blue light range, indicating the PL quenching and efficient sepration of photogenerated electrons and holes. The heterogeneous connection between GO and g-C_3_N_4_, with high conductivity of graphene, and high mobility of carriers in g-C_3_N_4_, effectively prevent electron-hole recombination in this structure^35–37^.

**Fig. 5:**
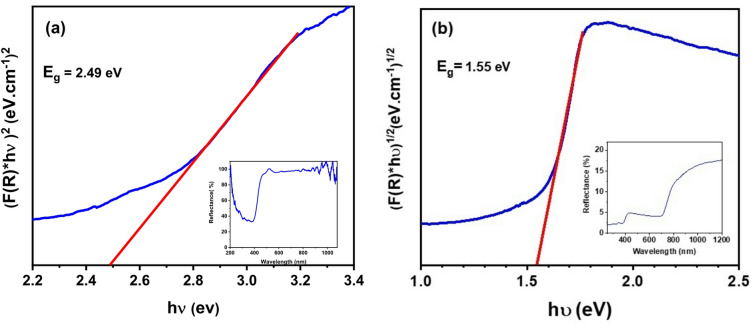
(**a**,**b**) Tauc plots of g-C_3_N_4_ and GO/g-C_3_N_4_ nanocomposite samples, the insets show reflectance spectra of samples.

The DRS analysis and Tauc plot of the g-C_3_N_4_ and GO/g-C_3_N_4_ nanocomposite samples are shown in Fig. 5a,b, respectively. To calculate the optical band gap of the g-C_3_N_4_ and GO/g-C_3_N_4_ nanocomposite samples, the Kubelka-Munk model and linear extrapolation of the Tauc plot were utilized. The following equation (Eq. 2) was employed for this calculation^38^:

2 $$\:{\mathrm{F}\left(\mathrm{R}\right)=\frac{{\upalpha\:}}{\mathrm{S}}=\frac{(1-\mathrm{R})}{2\mathrm{R}}}^{2}$$

where F(R) represents the Kubelka-Munk function, S is the scattering coefficient, α is the absorption coefficient, and R is the reflectance. The Tauc formula is a widely used method for determining the band gap of a material. It is expressed using Eq. (3).

3 $$\:{({\upalpha\:}\left(\mathrm{E}\right)\times\:\mathrm{h}\mathrm{v})}^{\mathrm{n}}=\mathrm{A}\left(\mathrm{E}-{\mathrm{E}}_{\mathrm{g}}\right)$$

where α is the absorption coefficient, E is the photon energy, E_g_ is the band gap energy, and A is a constant. The value of n depends on the type of transition, with *n* = 2 for direct transitions and *n* = 1/2 for indirect transitions^39,40^. In the present work, for GO, g-C_3_N_4_, and GO/g-C_3_N_4_ nanocomposite sample “n” is considered as ½, 2, and ½, respectively^41–44^. As can be seen in Fig. 5, the band gap value for the g-C_3_N_4_ sample was found to be 2.49 eV (498 nm), while for the GO/g-C_3_N_4_ nanocomposite sample, it was determined to be 1.55 eV (800 nm).

**Fig. 6:**
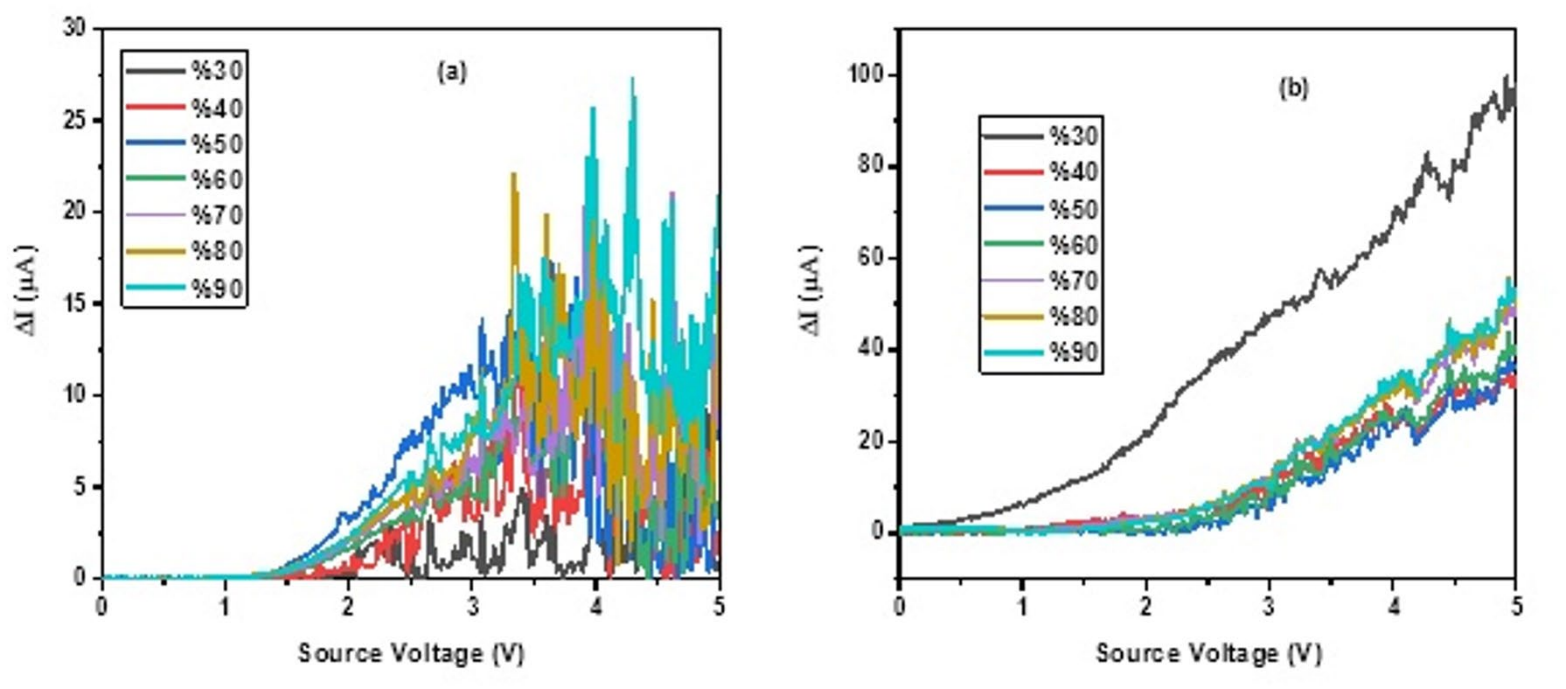
ΔI versus applied voltage at different humidity percentages for (**a**) g-C_3_N_4_ and (**b**) GO/g-C_3_N_4_ nanocomposite.

The humidity sensor response of the samples was studied at different humidity levels, ranging from ambient humidity (21%) – 90%. The sensing properties of the g-C_3_N_4_ and GO/g-C_3_N_4_ nanocomposite were measured as follows: The fabricated resistive sensors were first exposed to ambient humidity for 60 min to reach a stable baseline resistance. Then the sensors were exposed to different humidities in dark condition, the current-voltage curves under different RH levels and without light irradiation are shown in Fig. S1. We have used Eq. (4) to investigate the changes in current.

4 $$\Delta I = \left|{{\mathrm{I}}_{\mathrm{d}{\prime\:}\mathrm{H}}-\mathrm{I}}_{0}\right|$$

where $\:{\mathrm{I}}_{\mathrm{d}{\prime\:}\mathrm{H}}$ and I_0_ represent the electric current that passes through the sensor in the presence of determined RHs, and the current at ambient humidity (21%), respectively. The results are shown in Fig. 6. As can be seen in Fig. 6a, the g-C_3_N_4_ sensor did not show linear behavior in different humidity percentages. This behavior could be due to the absence of hydroxyl groups attached to the external surface of g-C_3_N_4_, so this material could not be considered as a sutable candidate for humidity sensor. Figure 6b shows that an increase in relative humidity led to a decrease in the current in the GO/g-C_3_N_4_ nanocomposite sensor. No significant changes were observed with respect to the humidity changes, except for 30% humidity. At 30% humidity, water molecules may form hydrogen bonds with each other and with the nanocomposite, leading to an increase in carriers. As the number of carriers increases, the electric current also increases. In general, neither of the two samples exhibited acceptable sensor behavior in conditions without light irradiation.

**Fig. 7:**
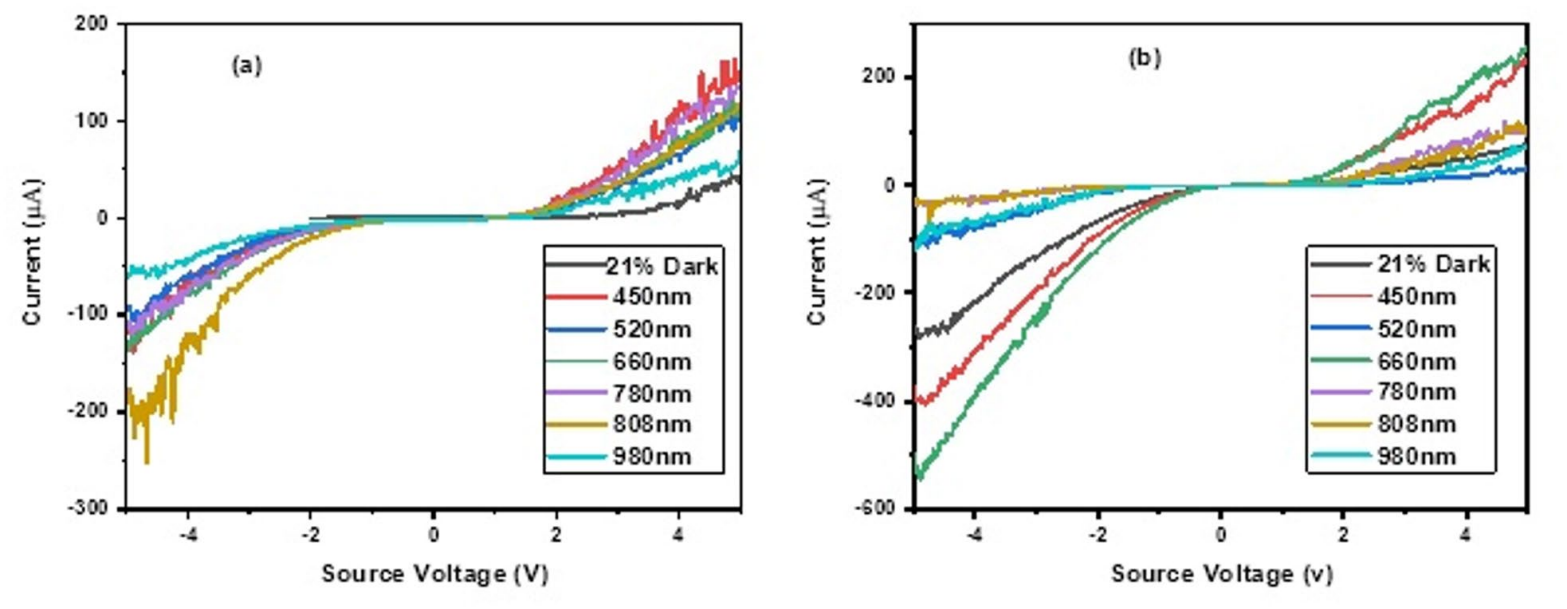
I–V curves of sensors under laser radiation with different wavelengths at room temperature and base humidity (21%) for (**a**) g-C_3_N_4_, and (**b**) GO/g-C_3_N_4_ nanocomposite samples.

Then the response of the sensors under different lasers has been studied and shown in Fig. 7. Figure 7a shows the I–V curves for the g-C_3_N_4_ nanosheet-based sensor under different laser light irradiations at the ambient conditions (RH = 21%). As can be seen in Fig. 7a, the highest current is observed under illumination with a wavelength of 450 nm. This result is due to the high photon absorption in this wavelength, which is in good agreement with the DRS analysis of the sample showing an absorption edge at about 450 nm. Photons with this wavelength can easily excite electrons in the conduction band and contribute to the generation of current^45^. The lowest current was recorded under irradiation at a wavelength of 980 nm. Photons with a wavelength of 980 nm have low energy, which is about 1.27 eV. For this reason, they may not be able to effectively excite electrons into the conduction band, which leads to a decrease in current.

Figure 7b shows the recorded current–voltage curves at different radiation wavelengths for the nanocomposite sample. The highest current is observed under irradiations at 660 nm and 450 nm, while the lowest current is observed under infrared irradiation (980 nm). Under light irradiation and ambient humidity (RH = 21%), irradiation at 450 nm and 660 nm in this nanocomposite caused an approximately four-fold increase in the current; at other wavelengths, the current changes under light irradiation were negligible. This phenomenon not only shows the importance of choosing the right wavelength to achieve suitable electrical properties of materials but also indicates the complex interactions between the material structure and environmental conditions. The impressive increase in current at the wavelength of 450 nm can be explained by the PL results of the sample, where, due to significant PL quenching with respect to g-C_3_N_4_ sample, the recombination of photoelectrons reduced and an increased current is observed.

**Fig. 8:**
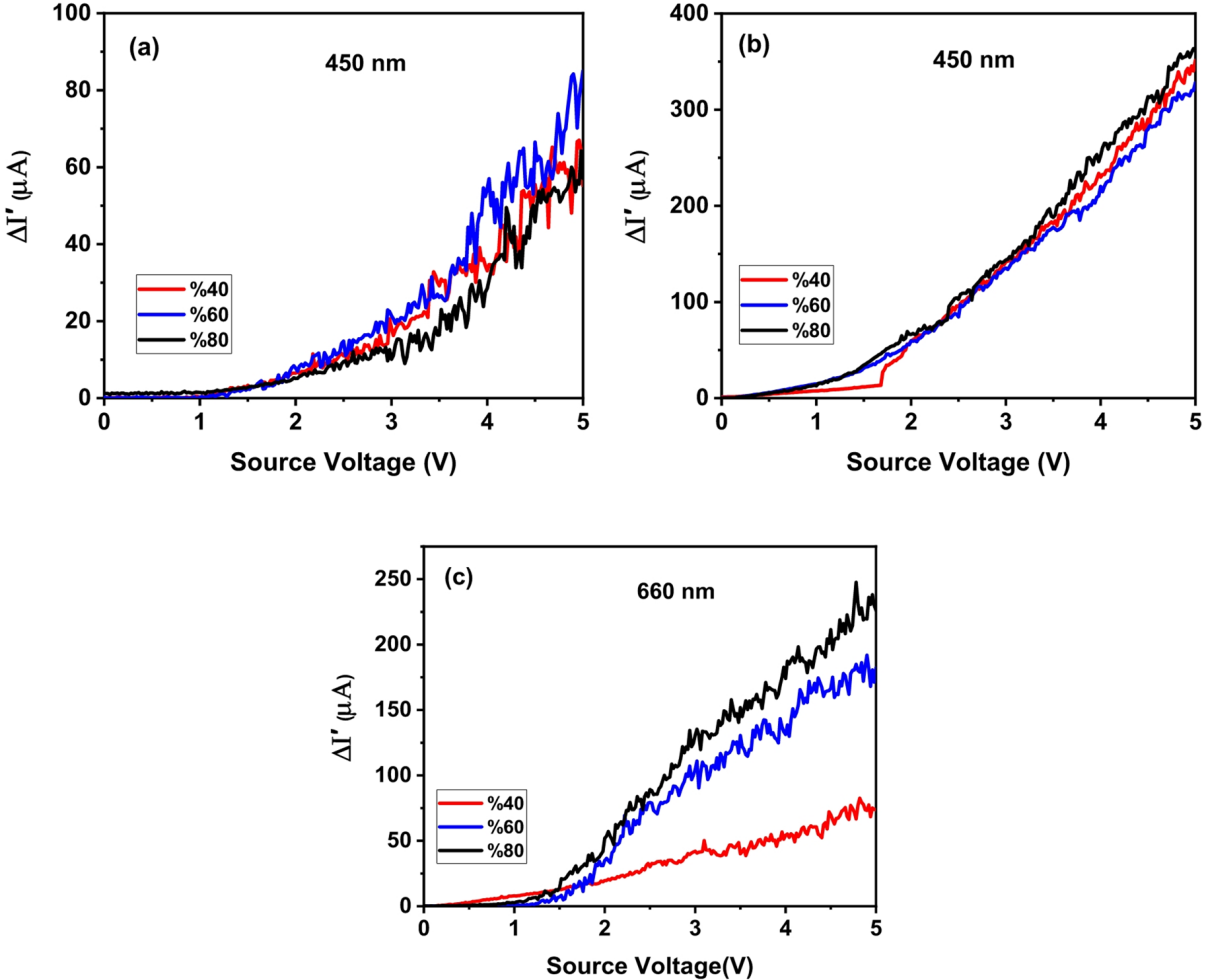
ΔI′ versus voltage in different humidities, (**a**) under illumination of laser with 450 nm wavelength for g-C_3_N_4_ sensor, (**b**) and (**c**) for GO/g-C_3_N_4_ sensor under illumination of lasers with 450 nm and 660 nm wavelengths, respectively.

Next, the current-voltage of sensors for different RHs, under different laser irradiations, with λ = 450 nm for g-C_3_N_4_, and 450 nm and 660 nm for GO/g-C_3_N_4_ nanocomposite were recorded, and reported in Fig. S2. Figure 8 illustrates the variation of ΔI′ in the g-C_3_N_4_ andnanocomposite-based photo-humidity sensor in response to the applied voltage at different humidity levels. Here, ΔI′ is defined as.

5 $$\Delta I' = \left|{\mathrm{I}}_{\mathrm{P}\mathrm{h}.\mathrm{H}}-{\mathrm{I}}_{0}\right|$$

where I_Ph, H_ represents the current under light radiation at various humidity percentages, and I_0_ is defined previously, in Eq. (4). Although we encountered an increase in the current for the 450 nm radiation at ambient humidity, by applying different RHs, the sensitivity to humidity changes was not significantly for both g-C_3_N_4_ and GO/g-C_3_N_4_ sensors (Fig. 8a,b). However, in the 660 nm radiation, with increasing the applied humidity, we see an increase in the optical current (Fig. 8c).

**Fig. 9:**
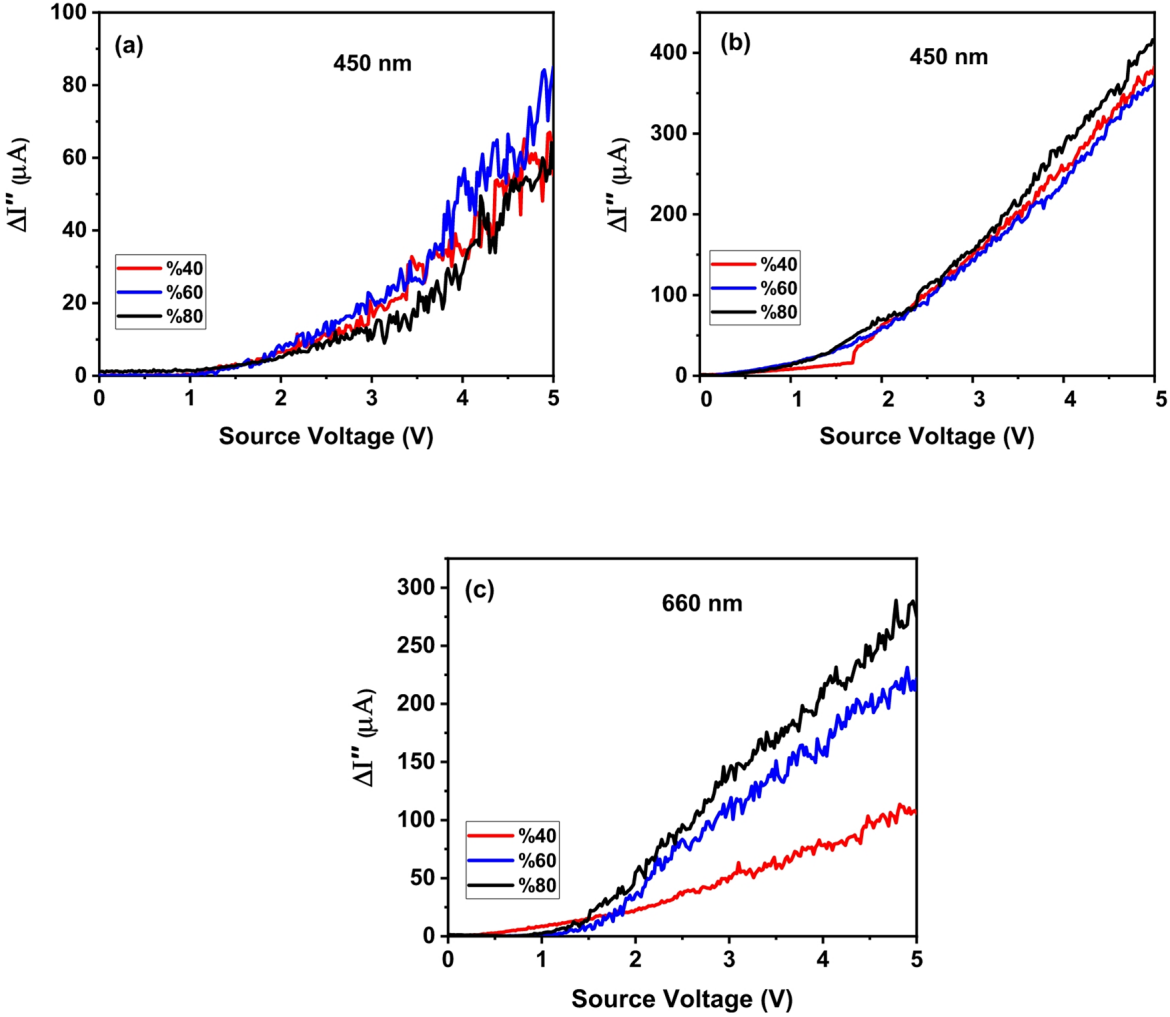
ΔI′′ versus voltage @ different humidities for (**a**) g-C_3_N_4_ under illumination with 450 nm, and (**b**) and (**c**) for GO/g-C_3_N_4_ under illumination with 450 nm and 660 nm wavelengths, respectively.

To exclusively investigate the role of light radiation at different RHs, the changes in current (ΔI″) versus applied voltage are shown in Fig. 8.

6 $$\Delta I'' = \left|{\mathrm{I}}_{\mathrm{P}\mathrm{h}{\prime\:}\mathrm{H}}-{\mathrm{I}}_{\mathrm{d}{\prime\:}\mathrm{H}}\right|$$

where ΔI″ is defined as the difference between the photocurrent at different RHs and under light irradiation (I_Ph_,_H_) and the current at same RH and dark condition (I_d_, _H_). It is clear that by increasing the RH, the photocurrent increases. While there is little change in both sensors in the 450 nm radiation (Fig. 9a,b), these changes are clearly evident at the wavelength of 660 nm for the GO/g-C_3_N_4_ sensor (Fig. 9c). The figure illustrates that under 450 nm irradiation, for g-C_3_N_4_ sensor, the ΔI″ do not follow a consistent or increasing trend compared to the dark current at the same humidity percentage (Fig. 9a). In contrast, for the GO/g-C_3_N_4_ nanocomposite sensor, an increase in the current was observed under 450 nm irradiation in the base humidity. However, by increasing the humidity level, no significant change in current concerning various RHs was detected (Fig. 8b and 9b). Conversely, under 660 nm irradiation, an increase in photocurrent was observed with increasing applied humidity (Fig. 8c and 9c).

**Fig. 10:**
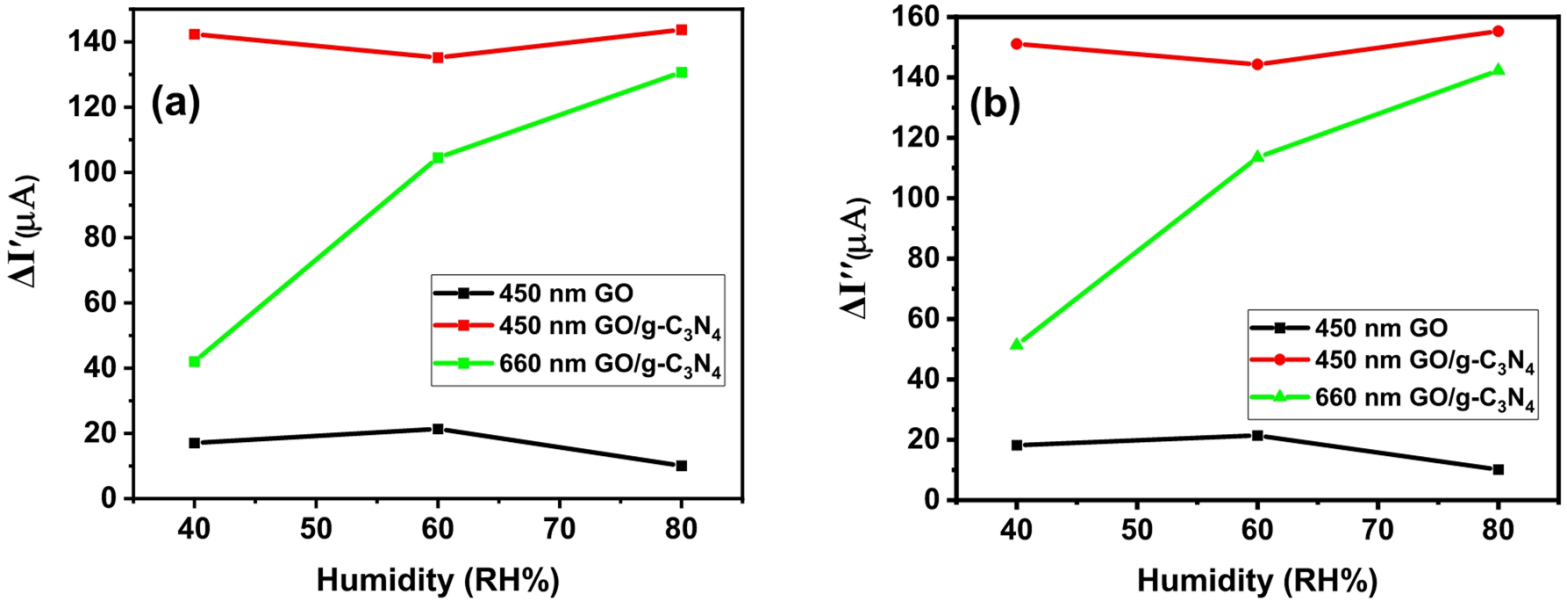
(**a**) ΔI′ and (**b**) ΔI′′ versus humidity percentages @ applied voltage of 3 V.

The current changes versus humidity levels of 40%, 60%, and 80% at the bias voltage of 3 V are shown in Fig. 10a,b. Under 660 nm irradiation, we observe a linear increase in current in the nanocomposite sensor, with rising humidity level. This increase in current at 660 nm, for both ΔI′ and ΔI′′, indicates the influence of the radiation wavelength on the light-activated performance of the GO/g-C_3_N_4_ nanocomposite sensor.

**Fig. 11:**
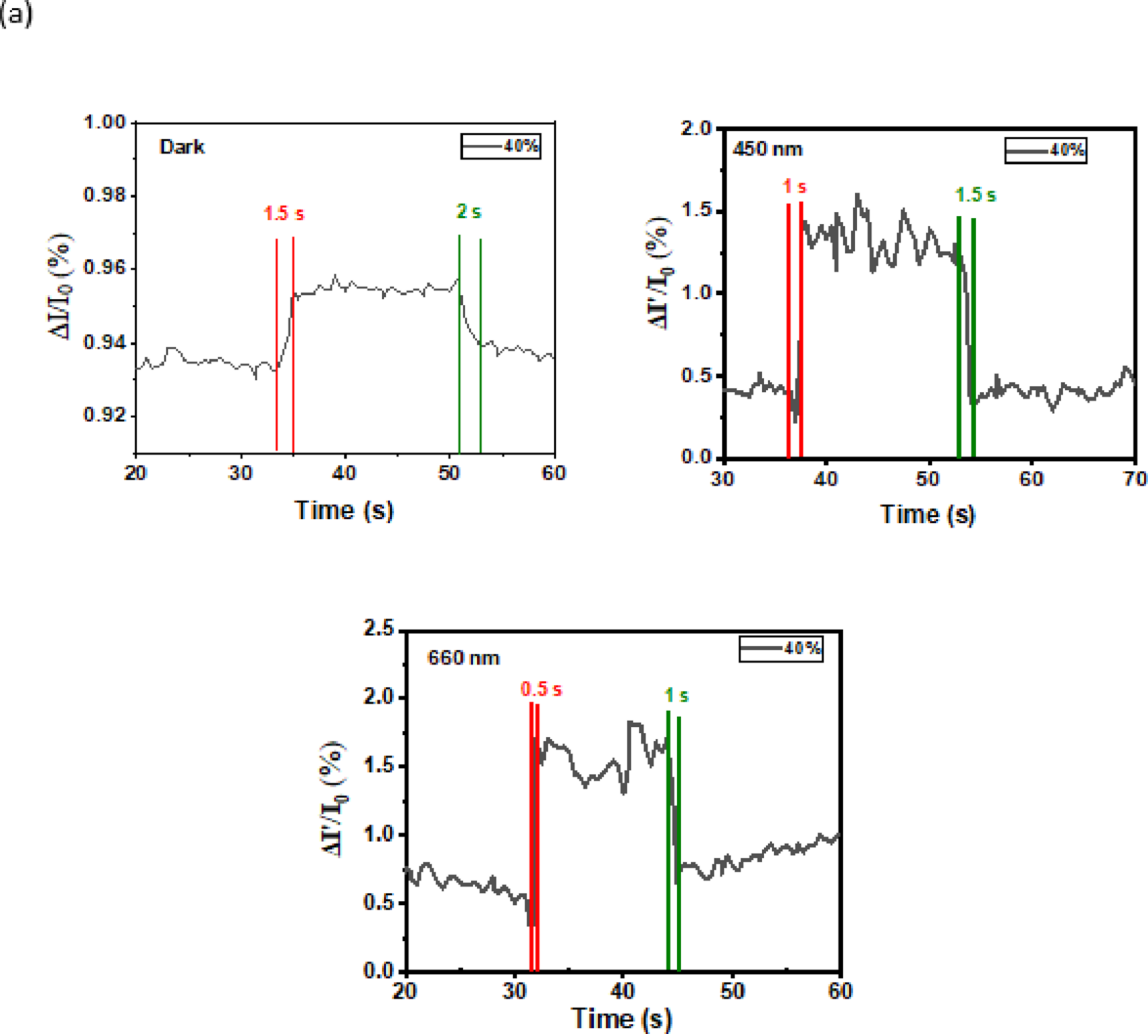

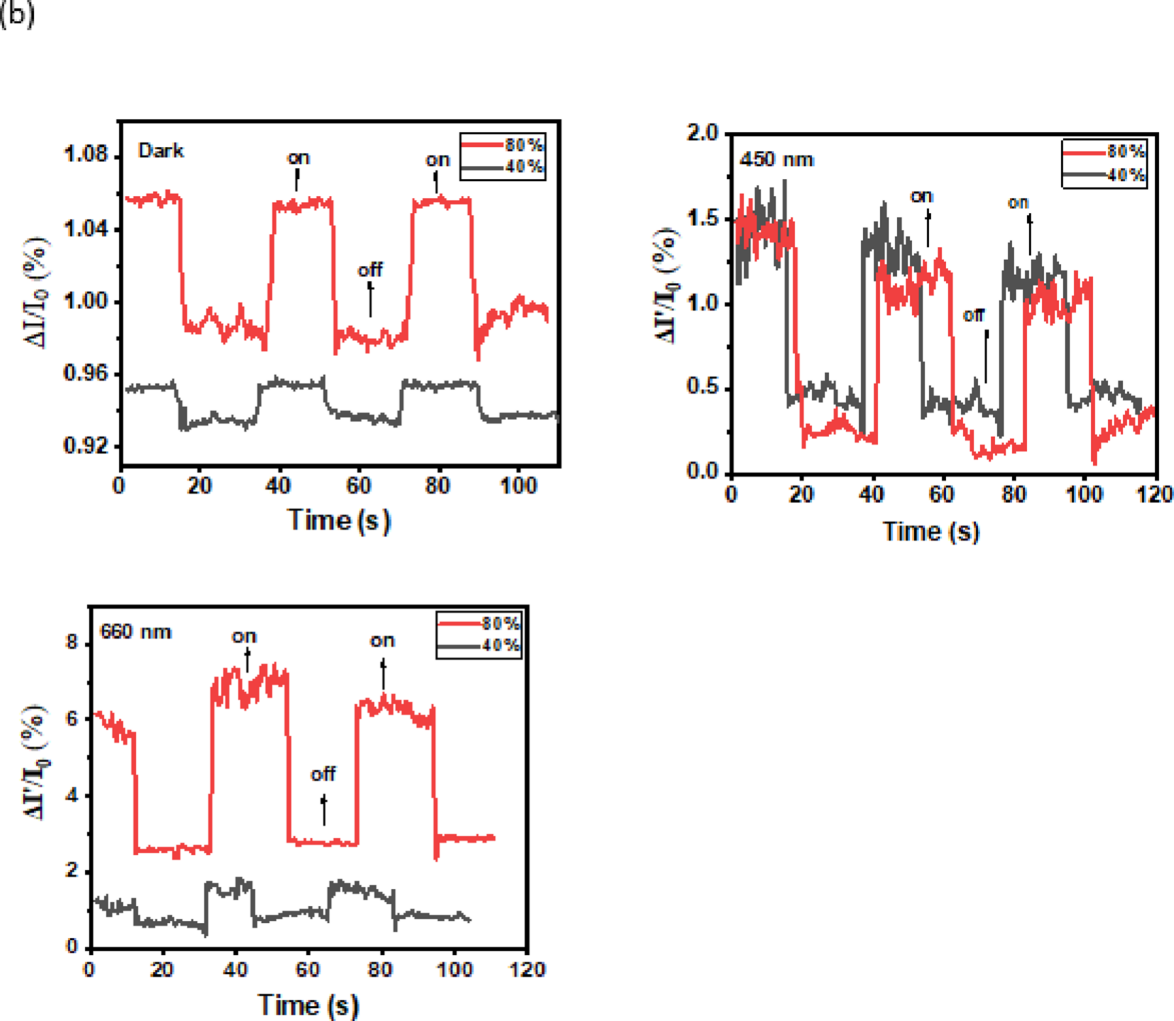
(**a**) Responsivity at 40% humidity, and (**b**) Reliability plots for RH 40% and 80% at dark condition, and illumination of the lasers with wavelengths of 450 and 660 nm.

Next, two key parameters in evaluating the performance and speed of humidity sensors, i.e. sensor response time and recovery time, have been examined. Response time measures the duration for a sensor to reach 90% of its steady-state output upon stimulus application, and recovery time, conversely, tracks the return to baseline (90% of initial value) after stimulus removal^46^. Figure 11a shows the relative change of current plots for the GO/g-C_3_N_4_ nanocomposite sensor for RH 40% at dark and under light irradiation at wavelengths of 450 and 660 nm. The calculated response times 1.5, 1, and 0.5 s were obtained for the mentioned cases, respectively, while the recovery times were 2, 1.5, and 1 s, respectively. The shortage response and recovery time for the composite sample was obtained at a wavelength of 660 nm and under misture. The shortage response and recovery time for the composite sample was obtained at a wavelength of 660 nm and under humidity. Furthermore, the Reliability of the nanocomposite humidity sensor has been examined under identical conditions and repeated measurements. This is a critical characteristic to ensure the accuracy and reliability of the data obtained from the sensors. The repeatability of the GO/g-C_3_N_4_ nanocomposite sensor at two different RHs (40% and 80%) at dark condition and under irradiation of lasers with wavelength of 450 and 660 nm is shown in Fig. 11b.

To the best of our knowledge, not much research has been done on light-activated humidity sensors, and this field has room for work and progress, especially as it could lead to the fabrication of humidity sensors with room operating temperature. Table 3 lists some recent work on light-activated humidity sensors. As shown in the table, our fabricated g-C_3_N_4_/GO nanocomposite demonstrates a reduced optical band gap compared with pristine g-C_3_N_4_, which facilitates improved charge - carrier generation and enhances humidity responsivity. Furthermore, faster response and recovery times, indicating improved adsorption/desorption kinetics due to the synergistic g-C_3_N_4_-GO interface and photo-activation effect.

**Table 3:** Comparison of of the current study results with the previously reported researchs on light-activated humidity sensors.

Sensor material	Band gap (eV)	Light radiation	Sensitivity	RH range (%)	Response/recovery times (s)	References
g-C_3_N_4_/GQDs	–	–	√	7–97	44/10	^21^
GQDs	–	–	√	7–97	27/80	^21^
Graphene/graphene oxide nanosheets	1.56	450 nm	√	30–80	1/1.3	^44^
ZnO nanowires	3.29	UV radiation	√	25–95	–	^47^
g-C_3_N_4_	2.27	450 nm	×	20–90	–	This work
GO/g-C_3_N_4_	1.55	Dark	×	21–90	1.5/2	This work
GO/g-C_3_N_4_	1.55	660 nm	√	20–80	0.5/1	This work

## Conclusion

In this study, the comparison of the light-activated humidity sensors based on g-C_3_N_4_ and GO/g-C_3_N_4_ nanocomposite has been investigated. At first the synthesis and characterization of both samples were studied. At dark condition, the results of humidity sensing for both sensor did not show significant changes or linear behavior. For the light-activated sensing study, although, the g-C_3_N_4_ sensor under irradiation at a wavelength of 450 nm passes higher current, due to insensitivity to different RHs and poor performance would not be a suitable candidate for humidity sensing application. Furthermore, the light-activated GO/g-C_3_N_4_ nanocomposite humidity sensor did not show linear behavior and acceptable performance at irradiation of 450 nm at different RHs. However, under irradiation at 660 nm with increasing the humidity level, an increase in current is recorded, which confirms that GO/g-C_3_N_4_ nanocomposite is a suitable sensor for this wavelength. These findings indicate the potential of the GO/g-C_3_N_4_ nanocomposite sensor for light-activated humidity sensing applications at room temperature, especially at 660 nm radiation or low-power light environments.

## Supplementary Information

Below is the link to the electronic supplementary material.

Supplementary Material 1

## Data Availability

The datasets used and/or analyzed during the current study are available from the corresponding author upon reasonable request.
