# Supplementary material for: Comparison of GO/g-C3N4 nanocomposite with g-C3N4 light-activated humidity sensors at room temperature
Source: Sci Rep. 2026 May 9;16:21345. doi: 10.1038/s41598-026-52607-3 (PMC13346496; doi:10.1038/s41598-026-52607-3)
Supplement: Supplementary file 1 — Supplementary Material 1 [file 41598_2026_52607_MOESM1_ESM.docx]

**Comparison of GO/g-C_3_N_4_ nanocomposite with g-C_3_N_4_ light activated humidity sensors at room temperature**

Nafiseh Tobeiha^1^, Nafiseh Memarian^1*^, Fatemeh Ostovari^2^

^1^ Faculty of Physics, Semnan University, Semnan

^2^ Department of Physics, Faculty of Science, Yazd University, Yazd

Figure S1: Current-voltage plots under different humidity levels without irradiation of light for (a) g-C_3_N_4_, (b) GO/g-C_3_N_4_ composite.

Figure S2: Current-voltage plots for different RHs, under different laser irradiations, with λ= 450 nm for g-C_3_N_4_ (a); 450 nm (b), and 660 nm (c) for GO/g-C_3_N_4_ composite.
